# Oral Metastasis of Metaplastic Breast Carcinoma in a Patient with Neurofibromatosis 1

**DOI:** 10.1155/2014/719061

**Published:** 2014-04-30

**Authors:** Ana Paula Molina Vivas, Luana Eschholz Bomfin, Clovis Antonio Lopes Pinto, Ulisses Ribaldo Nicolau, Fabio Abreu Alves

**Affiliations:** ^1^Stomatology Department, A.C. Camargo Cancer Center, Rua Professor Antonio Prudente 211, 01509-900 São Paulo, SP, Brazil; ^2^Stomatology Department, University of São Paulo, Avenida Professor Lineu Prestes 2227, 05508-000 São Paulo, SP, Brazil; ^3^Pathology Department, A.C. Camargo Cancer Center, Rua Professor Antonio Prudente 211, 01509-900 São Paulo, SP, Brazil; ^4^Clinical Oncology Department, A.C. Camargo Cancer Center, Rua Professor Antonio Prudente 211, 01509-900 São Paulo, SP, Brazil

## Abstract

Neurofibromatosis type 1 (NF1) has been associated with an increased risk for development of malignancy, especially malignant peripheral nerve sheath tumors. In addition, recently, literature has demonstrated an increased risk of breast cancer in women with NF1. The present paper shows a 53-year-old woman with NF1 who presented with metaplastic breast carcinoma and developed multiple metastases, including mandible. Furthermore, we reviewed the English literature, found 63 cases showing the association between NF1 and breast cancer, and added one more case. The present study demonstrated an important association between NF1 and breast cancer. Until the present time, there has been only one case of metaplastic breast carcinoma associated with NF1. Curiously, in our case the oral metastasis corresponded to sarcomatous component of metaplastic breast carcinoma.

## 1. Introduction

Neurofibromatosis type 1 (NF1) is an autosomal dominant genetic disorder with a prevalence of 1 in 3500 people. This fully penetrant condition is characterized by multiple café au lait spots, axillary and inguinal freckling, cutaneous neurofibromas, and iris Lisch nodules [1, 2]. Less common manifestations include plexiform neurofibromas, optic nerve, and other central nervous system gliomas, scoliosis, tibial dysplasia, and vasculopathy. Additionally, patients with NF1 have increased relative risk of developing malignant peripheral nerve sheath tumors, leukemia, rhabdomyosarcoma, gastrointestinal stromal tumors, phaeochromocytoma, and breast cancer [3–8].

Metaplastic breast carcinoma (MBC) is uncommon, accounting for less than 5% of breast carcinoma [9]. This tumor consists of a heterogeneous group of malignancies which may correspond to mixed epithelial and sarcomatoid components, as well as primary squamous cell carcinoma, or mixed adenocarcinoma and squamous cell carcinoma [10]. Interestingly, there is only one case of MBC affecting a NF1 patient previously reported in the English literature [11].

The present study presents a rare case of a patient with NF1 who developed MBC, which metastasized to oral cavity. In addition, the importance of investigating breast tumors in NF1 patients is emphasized.

## 2. Case Report

A 53-year-old woman was referred to the Stomatology Department, complaining of teeth mobility and a swelling in her mouth with 10 days of evolution. During the anamnesis, the patient denied any alcohol consumption and related that she had smoked for 18 years and quit 4 years ago. Her medical history included NF1 and left mastectomy 20 days previously due to metaplastic carcinoma.

**Figure 1:**
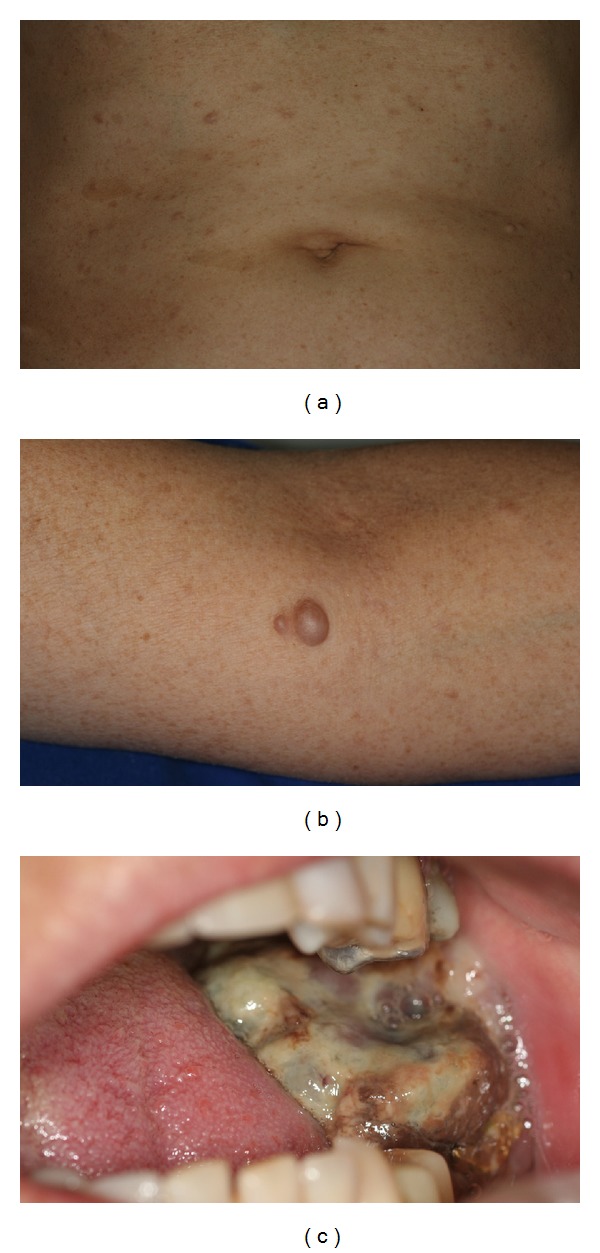
(a) Abdominal surface presenting café au lait macules and multiple cutaneous neurofibromas. (b) Upper member with cutaneous neurofibromas. (c) Intraoral view showing an extensive tumor with necrotic surface located on the left retromolar area.

On physical examination, the patient had multiple café au lait macules and cutaneous neurofibromas located on cervical, dorsal, abdominal, and upper members (Figures 1(a) and 1(b)). The intraoral examination revealed a large mass with necrotic surface in the left retromolar area, measuring approximately 5 centimeters, which caused important trismus (Figure 1(c)). The main diagnostic hypothesis was metastasis of MBC. In addition, under local anesthesia, the patient underwent incisional biopsy.

**Figure 2:**
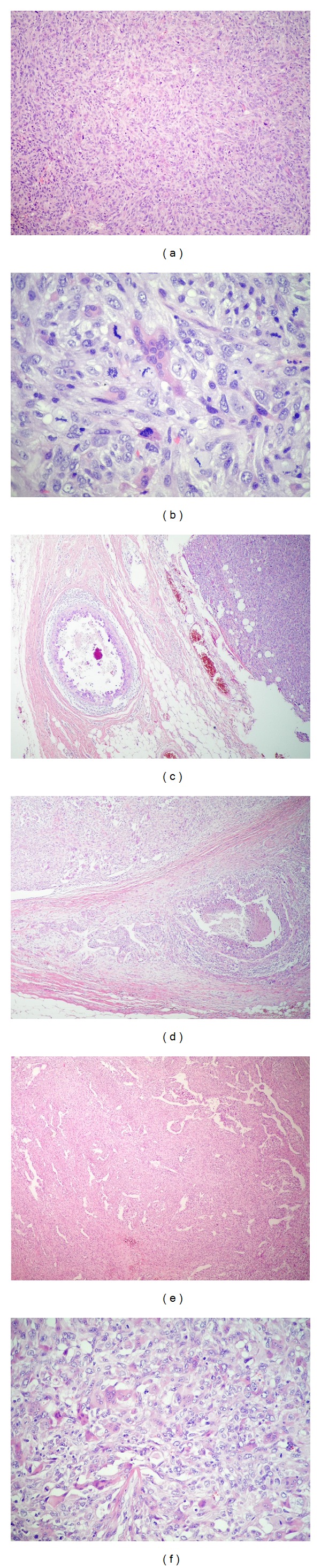
(a) Histological findings of malignant neoplasia of the oral cavity, showing atypical spindle cells with a storiform arrangement (H&E stain, ×40). (b) Areas with osteoclast-like cells (H&E stain, ×400). (c) Histological findings of metaplastic breast carcinoma with carcinoma* in situ* area beside the sarcomatous component (H&E, ×100). (d) Invasive ductal carcinoma (H&E, ×40). (e) Sarcomatoid pattern with hemangiopericytic area (H&E, ×40). (f) Sarcomatoid component with osteoclast-like cells (H&E, ×200).

The histopathological analysis of the oral cavity lesion revealed a malignant neoplasia with spindle cell pattern and areas with osteoclast-like cells (Figures 2(a) and 2(b)) suggestive of metastasis of MBC. Subsequently, the specimens of mastectomy were reviewed. The epithelial component of breast tumor exhibited areas of* in situ* (Figure 2(c)) and invasive ductal carcinoma (Figure 2(d)) and also areas with squamous differentiation. However, the major part of the tumor was composed of a sarcomatoid component with areas of hemangiopericytic pattern (Figure 2(e)) and others with osteoclast-like cells (Figure 2(f)).

**Figure 3:**
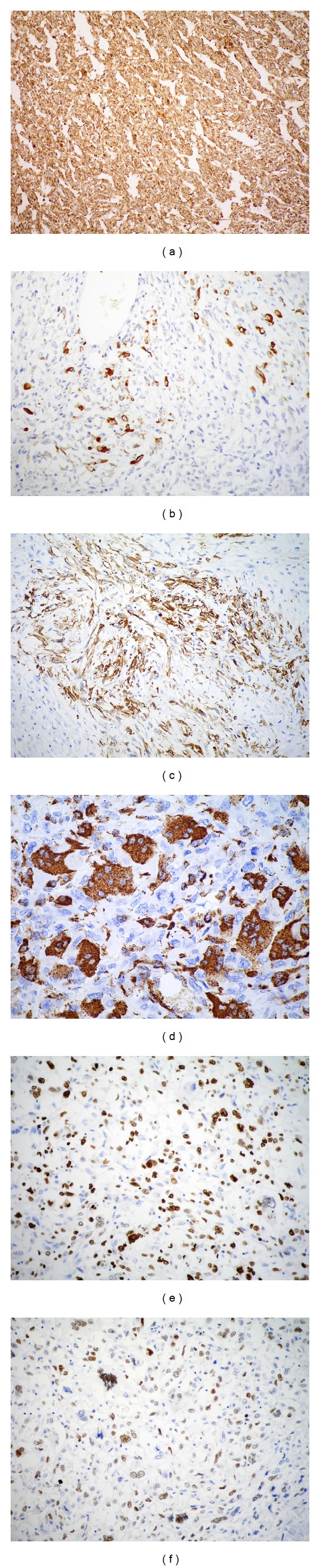
Immunoreactivity of metaplastic carcinoma. (a) Strong immunoreactivity for vimentin in the sarcomatous component. (b) Immunoreactivity for cytokeratin AE1/AE3 is present in few cells of the sarcomatous component. (c) Reactivity for smooth muscle actin in focal area. (d) Immunoreactivity for CD68 in osteoclast-like cells. (e) Nuclear immunoreactivity for Ki-67. (f) Expression of p53 (polymer-HRP detection system, biotin-free).

On immunohistochemical analysis, the breast tumor cells (Table 1) were negative for estrogen and progesterone receptors and c-Erb-B2 was only positive in carcinoma* in situ* area. Vimentin was positive in the sarcomatous component, while cytokeratin AE1/AE3 and p63 were seen in few cells of the same component. Furthermore, S-100 and smooth muscle actin were positive in focal areas and CD68 was positive in osteoclast-like areas. A strong nuclear positivity was found against p53 and Ki-67 antibodies (Figure 3). The immunohistochemical analysis of the mandibular biopsy specimen (Table 1) showed similar findings to those of the breast tumor, except for total negativity of p63 and S-100. Considering the clinical, histopathological, and immunohistochemical features, the diagnosis of oral metastasis of the sarcomatous component of MBC was confirmed.

The patient was referred to the Department of Clinical Oncology for evaluation. Computed tomography showed multiple lung and liver nodules and osteolytic lesion on the second costal arch. Moreover, all lesions were strongly suggestive of metastases. Chemotherapy with doxorubicin and ifosfamide was started but was interrupted due to pancytopenia. There was progression of the disease and the patient died 75 days after the diagnosis of oral metastasis.

## 3. Discussion

NF1 has been associated with cancer predisposition. The most common tumors are gliomas, malignant peripheral nerve sheath tumors, leukemia, and rhabdomyosarcoma [3, 20]. Although Brasfield and Das Gupta [12] reported in the 70s that 5 out of 54 women with NF1 developed breast carcinoma, only recently this association was recognized. Considering that breast cancer is already a common tumor in women, it would be difficult to know whether the coexistence of NF1 and breast cancer is a coincidence or a real predisposition. Sharif et al. [5] evaluated 304 women with NF1 and 14 had breast cancer (11 with infiltrating ductal carcinoma and 3 with infiltrating lobular carcinoma). Interestingly, these women had an early age of onset of breast cancer, with a median age of diagnosis of 44 years. Recently, Madanikia et al. [6] reviewed charts of 124 women with NF1 who were 20 years old or older and found 4 cases of breast cancer. Wang et al. [7] found 11 cases of breast cancer among a cohort of 76 women with NF1. Seminog and Goldacre [8] also showed a high risk of breast cancer, especially a threefold risk in women under 50. All these studies agree that women with NF1 are at higher risk for breast cancer than the general population, particularly when they are younger than 50 years old. Furthermore, these patients may have a delay in diagnosis since breast tumors may be misdiagnosed as NF1 manifestations [16, 17, 27]. In the present case, a 53-year-old woman with NF1 presented with a very aggressive breast cancer which metastasized to mandible, ribs, lung, and liver. Interestingly, on anamnesis, the patient related that she had undergone a mastectomy 20 days before, but she was being investigated due to breast nodule for 7 months.

We reviewed the English language literature and found 63 patients with NF1 who developed breast malignant tumors. Furthermore, most cases were ductal invasive carcinoma and less commonly lobular carcinoma (Table 2) [5–7, 11–31]. Interestingly, we found one well-documented case of MBC (carcinosarcoma) [11]. In the present case, the patient presented with metaplastic carcinoma with very scarce epithelial component, with the major part of the tumor composed of a sarcomatoid component. The oral lesion was exclusively formed by sarcomatoid fraction.

Metaplastic carcinoma is a very rare type of breast cancer, which accounts for less than 5% of breast carcinomas [9]. It is a poorly differentiated tumor characterized by coexistence of adenocarcinoma with areas of matrix producing, spindle-cell, sarcomatous, and/or squamous differentiation. The present case showed wide undifferentiated spindle cell elements; areas of hemangiopericytic pattern and abundant osteoclast-like cells were also observed. In contrast, the epithelial component was the minor part formed by invasive ductal carcinoma and carcinoma* in situ*. In addition, overexpression of c-Erb-B2 in metaplastic carcinoma is rare (4%), while estrogen and progesterone receptors are frequently negative. Consequently, this tumor is usually referred to as “triple negative” [32, 33]. Similar to most cases previously reported in the literature, the present case exhibited a triple-negative immunoprofile and also had a high histological grade, which caused many anomalous immunoexpressions, such as focal positivity to SMA, S-100 antibodies, and coexpression of vimentin and CK AE1/AE3 (1% of the cells) in the sarcomatous component. In addition, p53 and Ki-67 markers showed high proliferative rate in both breast and mandible tumors (Table 1; Figures 2 and 3).

Metastatic lesions comprise 1% of all oral cavity malignancies and usually represent the evidence of wide spread disease. According to the review of Hirshberg et al. [34] the most common primary sites for oral metastases in women are breast, female genital organs, kidney, and colorectum, while in men they are lung, kidney, liver, and prostate. Still, this review showed that the mandibular bone is more frequently affected than the oral soft tissues in a proportion of 2 : 1, with the mandible being the most common location and the molar area the most frequently involved. In our case, we believe that the oral metastasis occurred in the gingiva, since there was rapid growth of the necrotic lesion and absence of specific symptoms such as pain and paresthesia. In addition, computerized tomography showed only a tumor mass emerging from the mandible without significant bone involvement. Other clinical findings of our patient included lung, bone, and liver metastases, which are the main sites of metastatic MBC [35]. Similar to our case, McMillan and Edwards [13] reported a case of bilateral mandibular metastases of breast carcinoma in a 41-year-old woman with NF1. It is noteworthy that the patient was only 27 years of age when she underwent a right radical mastectomy for removal of a breast carcinoma. Differently from our case, the oral lesion presented as a lump on the right jaw with an intact mucosa covering and the authors believed that the initial site of localization was within the bone.

Despite the follow-up of patients with NF1 and breast cancer, the literature data are not clear. Brasfield and Das Gupta [12] observed that all 5 patients died within 5 years of the diagnosis of breast cancer. This fact led them to question whether neurofibromatosis could influence the prognosis of patients with cancer. Nevertheless, some authors correlated the poor prognosis with late diagnosis since breast tumors may be misdiagnosed as NF1 manifestations as commented before [16, 17, 27]. Considering the 64 patients, information about follow-up was found in 36 patients. Of these, 17 are alive, 16 dead of breast cancer, and 3 dead due to other causes (Table 2).

In summary, since breast cancer has been associated in the literature with NF1, affected patients require screening for breast tumors. Thereby, early identification of breast cancer is important for appropriate management and better prognosis of the disease. Interestingly, the case presented here is the second reported in the English language literature referring to an MBC involving a woman with NF1, along with the curious finding that there was metastasis of the sarcomatous component to oral cavity.

## Figures and Tables

**Table 1:** Immunohistochemical features observed in both carcinoma and sarcomatoid components and in both breast and mandibular tumors.

Antibody	Clone	Breast Cancer		Oral metastasis
		Carcinoma	Sarcomatous	
ER	SP1—DAKO	−	−	−

PR	PgR636—DAKO	−	−	−

C-erbB-e	Polyclonal—DAKO	(1+) invasive carcinoma	−	−
		(3+) carcinoma *in situ *	(0)	(0)

CK5	XM26 (mouse)—Neomarkers	−	−	−

CK14	LL002 (mouse)—Thermo Scientific	+ carcinoma *in situ *	−	−

CKAE1/AE3	AE1-AE3—DAKO	+	+	+
			1% neoplastic cells	1% neoplastic cells

p63	4A4—DAKO	−	+	−
			10% neoplastic cells	

p53	D0-7—DAKO	+	+	+
		20% neoplastic cells	40% neoplastic cells	80% neoplastic cells

Ki-67	MIB-1—DAKO	Proliferative activity	Proliferative activity	Proliferative activity
		50%	80%	90%

Vimentin	V9—DAKO	−	+	+

SMA	1A4—DAKO	−	+ focal areas	+ focal areas

Desmin	D33—DAKO	−	−	−

Myogenin	F5D—DAKO	−	−	−

Myo-D1	5.8A—DAKO	−	−	−

S-100	Polyclonal—DAKO	−	+ focal areas	−

CD68	KP1—DAKO	−	+ osteoclast-like cells	+ osteoclast-like cells

ER: estrogen receptor; PR: progesterone receptor; CK: cytokeratin; SMA: smooth muscle actin.

**Table 2:** Total of patients with NF1 who developed breast carcinoma considering only English language literature.

Authors	*N*	Breast cancer subtype	Age (years)	Follow-up (months)
Brasfield and Das Gupta [12]	5	Breast carcinoma*	1 patient 39, the others not informed	All dead within 60

McMillan and Edwards [13]	1	Spheroidal-cell carcinoma	27	Dead, 168

El-Zawahry et al. [14]	2	Lobular carcinoma Breast carcinoma*	40 70	Not informed

Zöller et al. [15]	2	Ductal carcinoma Ductal carcinoma	38 66	Dead 36 Dead 24

Nakamura et al. [16]	1	Scirrhous carcinoma	49	Dead 5

Murayama et al. [17]	1	Ductal carcinoma	66	Alive 8

Ceccaroni et al. [18]	2	Breast carcinoma* Breast carcinoma*	52 66	Not informed

Satgé et al. [19]	1	Ductal carcinoma	23	Alive 168

Güran and Safali [20]	2	Ductal carcinoma Ductal carcinoma	23 58	Not informed

Posada and Chakmakjian [21]	1	Lobular carcinoma	74	Alive 36

Walker et al. [22]	5	4 ductal carcinoma 1 lobular carcinoma	Mean age 46.4	Not informed

Natsiopoulos et al. [11]	1	Metaplastic carcinoma	60	Alive 30

Sharif et al. [5]	14	11 ductal carcinoma 3 lobular carcinoma	Mean age 43.5	5 died mean 66 3 died other causes 6 alive mean 54

Hasson et al. [23]	1	Ductal carcinoma	49	Not informed

Invernizzi et al. [24]	1	Ductal carcinoma	60	Alive 36

Alamsamimi et al. [25]	1	Ductal carcinoma	51	Alive 24

Hegyi et al. [26]	1	Malignant myoepithelioma	41	Not informed

Salemis et al. [27]	1	Ductal carcinoma	59	Alive 20

Bhargava et al. [28]	1	Ductal carcinoma	58	Alive 13

Takeuchi et al. [29]	1	Ductal and lobular carcinoma	69	Alive 6

Zhou et al. [30]	1	Ductal carcinoma	48	Alive 8

Madanikia et al. [6]	4	3 ductal carcinoma 1 unknown	Not informed	Not informed

Wang et al. [7]	11	10 ductal carcinoma 1 lobular and ductal carcinoma	Mean age 48.8	Not informed

Campos et al. [31]	2	Breast carcinoma* Ductal carcinoma	40 35	Dead Alive 24

Present case	1	Metaplastic carcinoma	53	Dead 3

Total	**64**			

*Subtype not informed.
